# Programme Science: a route to transformative change to improve population‐level impact for global HIV and sexually transmitted infections

**DOI:** 10.1002/jia2.26300

**Published:** 2024-07-10

**Authors:** Marissa L. Becker, Maryam Shahmanesh, Sevgi O. Aral

**Affiliations:** ^1^ Institute for Global Public Health University of Manitoba Winnipeg Manitoba Canada; ^2^ University College London London UK; ^3^ Africa Health Research Institute Durban South Africa; ^4^ Centres for Disease Control and Prevention Atlanta Georgia USA

Tremendous progress has been made in managing the global HIV epidemic, both in terms of reducing new HIV acquisitions and in improving coverage of treatment for people living with HIV [1]. However, these gains have not been achieved everywhere, nor equitably for all people [2, 3]. In a context where funding is increasingly limited, we need to progress faster, better and more efficiently to have the greatest impact on both the HIV epidemic and other sexually transmitted and blood‐borne infections (STBBIs) globally [3, 4]. How do we achieve this? There is a need for transformative action for science‐led HIV and STBBI programming in order to reduce inequities, improve outcomes and realize the global goal of “leaving no one behind.”

Advances in biomedical, behavioural and social sciences have led to many new and effective innovations, for example in HIV diagnostics, treatment and prevention and through approaches such as community mobilization, decentralized care and structural interventions. While these tools and approaches have been implemented, too often they have proven ineffective on their own in reducing HIV acquisitions [5, 6, 7, 8]. Important questions remain as to whether, and how, these interventions can lead to population‐level impact [9, 10]. In addition, there are many comprehensive prevention programmes with good coverage but which have not resulted in population‐level impact, often as a result of not reaching the right populations, in the right places, at the right time or with the right programme components and intensity [11, 12, 13, 14, 15, 16]. In order to maximize population‐level impact by reducing inequities in programme coverage and health outcomes, science which is embedded in programmes is needed, which generates new knowledge that can be applied by programmes to ensure their progress in achieving impact in a timely manner.

Knowledge generation, learning and monitoring are an integral part of programmes but what is often missing is an organized and systematic process of gathering, analysing and using this knowledge in a continual and iterative manner to not only inform and optimize programmes but also to generate new knowledge which is generalizable and transferable to other contexts, and which can move scientific knowledge forward. This requires transdisciplinary approaches which embrace, rather than control for the complexity within public health [17]. Programme Science is an approach to public health programming and research that aims to improve the design, implementation and monitoring of public health programmes through the systematic application of theoretical and empirical scientific knowledge that is generated through programme‐embedded research and learning process [18, 19]. Programme Science is concerned with the totality of a public health programme and its context. It emphasizes getting research out of practice [20], generating new hypotheses and supporting knowledge back into practice. Acting as an integrative framework for both public health programming and research, it is defined by an iterative process whereby empirical and situated knowledge derived from programmes drives scientific inquiry, which then produces further evidence that is incorporated into programming for service optimization and population‐level impact, while also generating knowledge [21].

Programme Science integrates key programmatic *spheres of practice*—strategic planning, programme implementation and evaluation—with complementary and evolving *spheres of knowledge* [21]. These spheres of knowledge are illustrated by the papers in this supplement. They include understanding local epidemiology, defining the packages of interventions, designing ongoing and iterative monitoring to evaluate the programme and adapt the programme theory of change alongside the changes in epidemiology [15]. This is achieved by embracing the complexity of a public health programme and placing the communities most affected by the epidemic at the centre of the research and practice [22]. Programme Science is equity‐focused; it is concerned with identifying and prioritizing populations who will benefit most from programme activities and services, and allocating resources such that programme activities are accommodating the needs of prioritized populations [23].

To achieve population‐level impact, programmes must optimize the effective coverage of their component interventions such that the proportion of prioritized populations experiencing positive outcomes from programme services is maximized. Using Programme Science principles, McClarty et al. developed the Effective Programme Coverage Framework [24], a novel practice‐based tool for embedding rapid and iterative research and learning into HIV and STBBI programmes. The development of this framework is a part of a larger global HIV/STI Programme Science Initiative. This initiative brings together a network of global leaders and experts in STBBI prevention research, programming and policymaking and has informed the development of this supplement.

This supplement synthesizes work from various global contexts, including academics, programme managers, community, government leaders and funders. The papers in this supplement build upon earlier work of Programme Science and the Effective Programme Coverage Framework and present examples in which Programme Science is operationalized in different contexts. The supplement begins with a viewpoint, in which Reid et al. [25] highlight PEPFAR's perspective on challenges in improving programme coverage for the most marginalized populations. The authors articulate how Programme Science can help to understand practice gaps by generating new, context‐specific knowledge that is simultaneously generalizable to different settings. They emphasize that “… a shift in how programmes inform science and how and when science is used to shape its programmes” is required to achieve PEPFAR's goals, and doing so will necessitate enabling environments that support science and innovation.

Ramesh BM et al. [26] focus on the challenges with programme coverage gaps, presenting data from a sub‐national HIV epidemic appraisal in Kenya, which illustrates significant geographic heterogeneity in disease burden and prevalence by population. Aligning with a Programme Science approach, the authors clearly demonstrate how their novel approach to epidemic appraisals contributes to and informs more efficient and precise programming for prevention.

The next three articles illustrate the centrality of community voices in a Programme Science approach and, highlight the imperative for researchers to have greater accountability to local communities. Together, these papers articulate what is required to shift the paradigm to truly leave no one behind. Thomas et al. [27] argue that we need to explicitly centre key population communities and to recognize and utilize their expertise and knowledge through meaningful involvement in the development of prevention strategies. Lauer et al. [28] provide examples of how communities can engage meaningfully and systematically through community‐led monitoring. They share examples of how timely and contextually relevant knowledge and data generated through community‐led monitoring can, and should, be used to design solutions to locally identified challenges with programme delivery and coverage. Finally, Garcia et al. [29] illustrate the importance of academic engagement with communities. Using the example of a Human papillomavirus (HPV) self‐testing project, they demonstrate the integration of science within public health programming, focusing on the critical need for accountability to communities. The authors demonstrate how a Programme Science approach, which bridges the common disconnect between researchers and communities, can ensure that researchers are responding to communities’ needs and can facilitate the co‐creation process of knowledge with communities.

Hargreaves et al. [30] further the discussion about the opportunities that researchers have through Programme Science in understanding and measuring programmatic impact. The authors discuss how traditional research often focuses on managing and controlling for real‐world complexity with analytic approaches such as randomization and principles such as standardization and fidelity. They acknowledge that these approaches and principles are often quite limiting for programmes embracing and including the complexity of public health challenges in the real world. This highlights the need for evolving rigorous Programme Science approaches to conduct feasible, useful, real‐world‐relevant, and informative research and present how, through embedded science, research can be generated to strengthen programme impact, and ultimately improve health outcomes and equity.

The next four papers provide concrete examples of embedded research from Zambia, Zimbabwe, Nigeria and Kenya. Sikazwe et al. [31] present an application of a Programme Science cycle in Zambia, illustrating how changes in the implementation of PrEP led to hugely improved coverage and enabled the scalability of the programme. In order to improve PrEP coverage and to ensure that they reached the right population, their programmes moved to a more decentralized, venue‐based PrEP service delivery approach. This enabled increased uptake, decreased inequities in access and improved coverage across key population groups. Cowan et al. [32] and McClarty et al. [33] then illustrate the iterative nature of Programme Science through representation of the second and third spheres of Programme Science—programme implementation and programme monitoring and evaluation—with data then cycling back to inform programme strategy (first sphere). Cowan et al. [32] present two case studies from Zimbabwe which describe different approaches to programme optimization for a priority population, sex workers, to improve impact. Case study one is an example of “implementing better” through microplanning, a process that decentralizes outreach management and planning to peer outreach workers. Case study two is an example of “implementing differently” through the re‐orientation of the HIV prevention programmes to reach those at the highest risk of acquiring HIV. These revisions to programme strategy led to improved coverage, particularly for those with the highest need. Through embedded research, they were able to generate important evidence to inform critical, and nuanced decisions for refinement to programme strategy and implementation. Using routine data in a systematic way to implement better and implement differently as illustrated by Cowan et al. is transferable to inform scalability and intensification of sex worker programmes globally for population impact.

McClarty et al. [33] and Bhattacharjee et al. [34] illustrate the application of the Effective Programme Coverage framework to identify gaps in programme coverage and generate insights to inform solutions. Using survey data, McClarty et al. generate coverage cascades which highlight critical coverage gaps across key populations in Nigeria, clearly underscoring the non‐linear movement across the cascades. They demonstrate how these data can be used to optimize programmes locally in Nigeria as well as creating wider knowledge which can be translated to other settings. Thus, this approach can help provide much more nuanced understanding of epidemics and the evolution of epidemics which necessitate dynamic programme responses. Bhattacharjee et al. [34] use data from an expanded Polling Booth Survey conducted in partnership with key population communities in Kenya. Using the Effective Programme Coverage framework allowed the team to both identify and quantify gaps in programme coverage as well as generate drivers and contributors to these gaps. In addition, this work led to the generation of new research questions for future enquiry. These two papers demonstrate why coverage is important and how the application of a Programme Science approach to use and analyse routine programme data can help examine coverage gaps and address inequities.

Together these papers, written by researchers and scientists, programme managers, policymakers and community, present how across contexts, embedded research through a Programme Science approach can generate new knowledge and insights, while addressing community concerns. In addition, these papers illustrate how a Programme Science approach can help to overcome some of the challenges in the translation of traditional research into real‐world population‐level impact.

Future directions for Programme Science include advancing research methodologies and adopting newer interventions and approaches to allow for improved ways to measure these complexities and understand context [35]. As epidemics evolve, we need to be prepared to observe and understand the changes in the HIV and STBBI epidemics in real‐time so that we generate the necessary knowledge required for programmes to evolve and respond. As we develop greater experiences of applying a Programme Science approach across contexts, we hope to create typologies of contexts and a scientific understanding of how HIV and STBBI epidemics evolve across different types of contexts, especially in the setting of different types of programming. Ultimately, through embedded research, we can not only optimize programmes locally but advance our scientific understanding of how infectious disease epidemics evolve in relation to public health programming and how to generalize some of the knowledge created so we can learn, and address existing and emerging epidemics. Responding to the need for transformative action requires a critical shift in how we fund, design, implement, report on and use research to advance science for population impact.

## COMPETING INTERESTS

The authors declare no competing interests.

## AUTHORS’ CONTRIBUTIONS

The authors contributed equally to this work. MLB drafted the editorial with inputs from MS and SOA. All authors further revised the editorial and reviewed and approved the final version.

## FUNDING

The publication of this supplement was supported by the Bill & Melinda Gates Foundation.

## Data Availability

Data sharing is not applicable to this article as no datasets were generated or analysed during the current study.
